# Comparing In-Person, Standard Telehealth, and Remote Musculoskeletal Examination With a Novel Augmented Reality Exercise Game System: Pilot Cross-Sectional Comparison Study

**DOI:** 10.2196/57443

**Published:** 2025-02-05

**Authors:** Richard Wu, Keerthana Chakka, Sara Belko, Ninad Khargonkar, Kevin Desai, Balakrishnan Prabhakaran, Thiru Annaswamy

**Affiliations:** 1 The University of Texas Southwestern Medical Center, Dallas, TX, United States; 2 Johns Hopkins Hospital, Baltimore, MD, United States; 3 Northwestern Medicine, Chicago, IL, United States; 4 Sidney Kimmel Medical College at Thomas Jefferson University, Philadelphia, PA, United States; 5 Department of Computer Science, The University of Texas at Dallas, Richardson, TX, United States; 6 Department of Computer Science, The University of Texas at San Antonio, San Antonio, TX, United States; 7 Penn State Health Milton S. Hershey Medical Center, Penn State University College of Medicine, Hershey, PA, United States

**Keywords:** physical examination, telemedicine, tele-health, virtual care, virtual health, telerehabilitation, augmented reality, AR, game, simulation, digital world, virtual environment, motion, strength, force, musculoskeletal, remote examination, exercise, physical examinations, telehealth, cross-sectional, VIRTEPEX, patient, exergame

## Abstract

**Background:**

Current telemedicine technologies are not fully optimized for conducting physical examinations. The Virtual Remote Tele-Physical Examination (VIRTEPEX) system, a novel proprietary technology platform using a Microsoft Kinect-based augmented reality game system to track motion and estimate force, has the potential to assist with conducting asynchronous, remote musculoskeletal examinations.

**Objective:**

This pilot study evaluated the feasibility of the VIRTEPEX system as a supplement to telehealth musculoskeletal strength assessments.

**Methods:**

In this cross-sectional pilot study, 12 study participants with upper extremity pain and/or weakness underwent strength evaluations for four upper extremity movements using in-person, telehealth, VIRTEPEX, and composite (telehealth plus VIRTEPEX) assessments. The evaluators were blinded to each other’s assessments. The primary outcome was feasibility, as determined by participant recruitment, study completion, and safety. The secondary outcome was preliminary evaluation of inter-rater agreement between in-person, telehealth, and VIRTEPEX strength assessments, including κ statistics.

**Results:**

This pilot study had an 80% recruitment rate, a 100% completion rate, and reported no adverse events. In-person and telehealth evaluations achieved highest overall agreement (85.71%), followed by agreements between in-person and composite (75%), in-person and VIRTEPEX (62.5%), and telehealth and VIRTEPEX (62.5%) evaluations. However, for shoulder flexion, agreement between in-person and VIRTEPEX evaluations (78.57%; κ=0.571, 95% CI 0.183 to 0.960) and in-person and composite evaluations (78.57%; κ=0.571, 95% CI 0.183 to 0.960) was higher than that between in-person and telehealth evaluations (71.43%; κ=0.429, 95% CI −0.025 to 0.882).

**Conclusions:**

This study demonstrates the feasibility of asynchronous VIRTEPEX examinations and supports the potential for VIRTEPEX to supplement and add value to standard telehealth platforms. Further studies with an additional development of VIRTEPEX and larger sample sizes for adequate power are warranted.

## Introduction

Telemedicine has seen increased utilization in recent years, especially during the COVID-19 pandemic [1]. An important advantage of telemedicine is flexibility, which has led to its incorporation into various medical specialties, including radiology, psychiatry, dermatology, neurology, and cardiology [2]. Telemedicine can be useful for providing health care in difficult-to-reach locations, such as global health settings [3], rural or wilderness areas [4], disaster scenarios [5], and even outer space [6]. Many of these applications use video conferencing technology combined with peripheral examination devices, such as electronic stethoscopes, tele-opthalmoscopy cameras, video otoscopes, tele-dermatoscopes, digital endoscopes, electronic scales, smartphones, and wearable devices [2]. In the field of rehabilitation medicine, telecommunications technology has enabled telerehabilitation facilitating remote patient interactions [7]. Examples of telerehabilitation applications include replacing in-person visits for chronic lower back pain evaluation and management [8], providing remote synchronous treatment interventions for musculoskeletal conditions [9], and implementing asynchronous rehabilitation programs following total knee replacement surgery [10].

However, existing telemedicine and telerehabilitation platforms have limitations. One major limitation is that physical examinations are difficult to conduct through virtual platforms, as most telemedicine systems are limited to video and audio transmission. Consequently, many physical examination components, including auscultation [11], palpation [2], and manual strength assessments [12] cannot be easily performed through video or audio alone. These assessments may require either another provider at the remote site to assist with physical examination [2] or a peripheral device to collect biometric data. For musculoskeletal examinations, health care providers using existing telemedicine systems may attempt to remotely evaluate patients’ range of motion and concentric and eccentric strength by asking them to move a joint or extremity across its full range of motion, raise a limb against gravity, or lift objects of known weight [12]. However, these approaches are limited compared to in-person musculoskeletal examinations and may not always provide reliable information for a comprehensive tele-physical assessment (TelePA) by health care providers.

In recent years, emerging technologies have shown promise in improving reliability and accuracy of TelePA. Wearable biometric sensors can facilitate remote evaluation by transmitting spatiotemporal position, speed, acceleration, gait, force, and haptic data in real time [13-16]. Virtual and augmented reality technologies can immerse patients in simulated environments [13] and facilitate body ownership in virtual settings [17], thereby leading to greater patient motivation, engagement, and psychosocial benefits during telerehabilitation [18]. Some TelePA systems utilize motion-sensing 3D sensors, such as the Microsoft Kinect system [19], which is a portable, low-cost motion analysis system equipped with RGB+Depth (RGB-D) camera technology [20]. The Kinect system has been studied for various medical applications that involve examining upper and lower extremity function, including assessment of patient movement and function [21], detecting patient walking [22], performing gait analysis [23], assessing balance [24], and mobility monitoring in patients with Parkinson’s disease [25].

## Methods

### Overview

This pilot study aimed to expand the scope of telemedicine and telerehabilitation by assessing the feasibility of the novel Virtual Remote Tele-Physical Examination (VIRTEPEX) system, which combines an augmented reality computer game environment with the Microsoft Kinect system to track motion and estimate force during gameplay [26]. The VIRTEPEX system can be operated asynchronously to evaluate motion and strength and has the potential to augment TelePA during or between telemedicine visits.

### VIRTEPEX Game System

**Figure 1.**
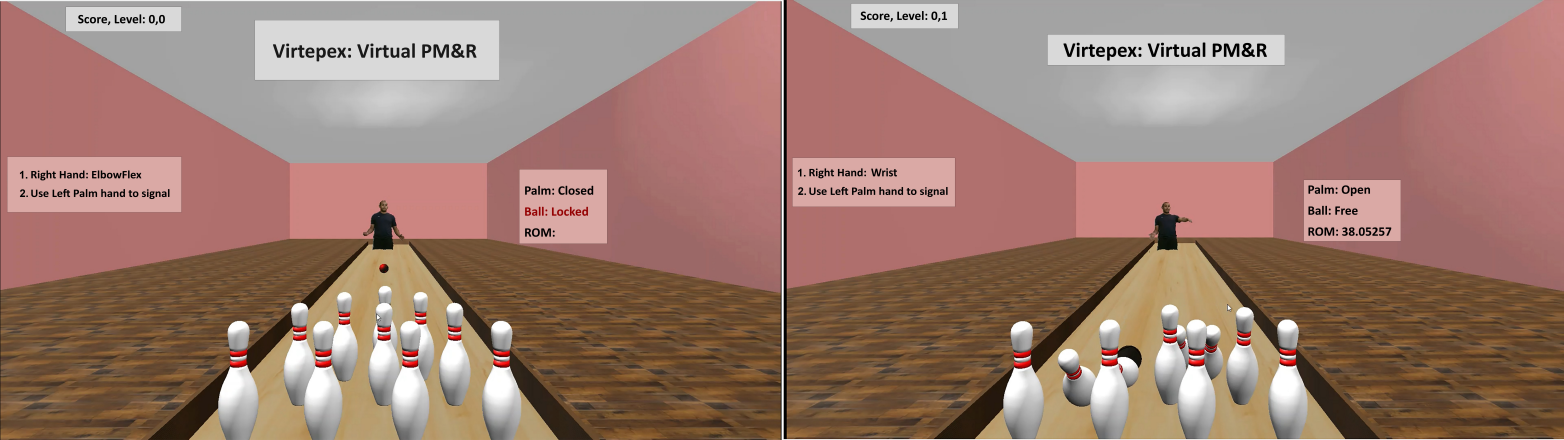
The simulated bowling computer game interface [26]. PM&R: Physical Medicine and Rehabilitation. ROM: Range of Motion

**Figure 2.**
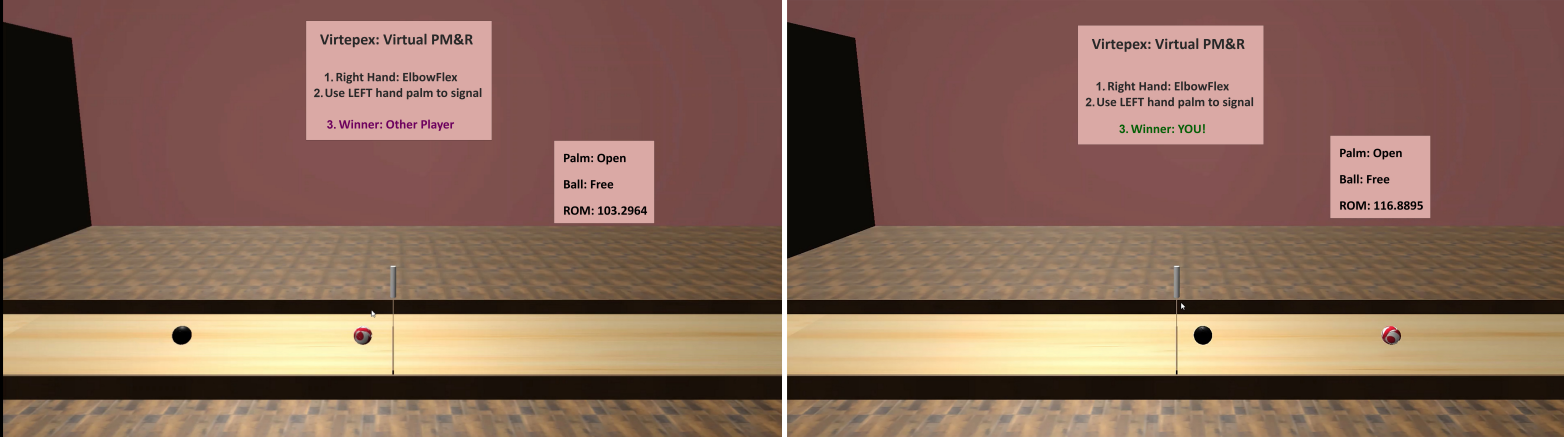
The simulated bowling ball collision animation as viewed by the health care provider for asynchronous remote evaluation of patient’s strength [26]. PM&R: Physical Medicine and Rehabilitation. ROM: Range of Motion

VIRTEPEX is a proprietary technology platform that uses the Microsoft Kinect (version 2) RGB-D camera [20] and machine learning software [27]. The technical specifications of VIRTEPEX have been previously published [26]. VIRTEPEX uses noninvasive motion tracking and inverse dynamics to estimate forces for 4 joint movements: shoulder abduction, shoulder flexion, elbow flexion, and wrist extension. As part of the user experience, patients perform the 4 joint movements while playing an augmented reality bowling computer game (Figure 1), which was rendered using Unity LTS (version 2018.4; Unity Technologies). For each joint movement completed, the patient’s strength determines the momentum of a virtual bowling ball for each completed joint movement. VIRTEPEX records and transmits force estimates for each joint movement to a health care provider, who can asynchronously assess the patient’s strength. During asynchronous evaluation, the health care provider performs the same 4 joint movements through VIRTEPEX; for each joint movement performed by both the patient and provider, VIRTEPEX synthesizes a comparative animation with two virtual bowling balls colliding into one another (Figure 2). In the animation, the momentum of one ball corresponds to the patient’s strength for the joint movement, while the other corresponds to the provider’s strength. A virtual midline marks the location where the balls would collide if both users applied equal strength; the provider visually estimates the distance between the bowling balls’ collision point and the midline to subjectively estimate the difference in strength between a patient and the provider. In other words, if the balls collide further away from the midline and closer to the patient, the patient’s joint movement strength is judged to be weaker compared to the provider.

### Study Sample

This cross-sectional pilot study included patients with impaired upper extremity movements due to any condition causing shoulder, elbow, or wrist pain and/or weakness, with the aim of evaluating movement strength. Adult patients with history of pain and/or weakness in these areas were recruited from a Veterans Affairs hospital’s outpatient physical medicine and rehabilitation clinic over one month. Patients scheduled for a therapy (ie, physical, occupational, or kinesiological) for any of the above symptoms were approached for participation; of 15 patients who nitially agreed to participate, 3 patients did not show up for their scheduled research visit, leaving 12 patients who were finally recruited. Of these, 10 participants had unilateral upper extremity pain and/or weakness while the other 2 participants had bilateral issues, resulting in a total of 14 evaluations. This number is similar to that previously reported for comparable clinical studies of telerehabilitation technology [7]. Before formal evaluations, participants and evaluators received training on VIRTEPEX and were allowed to interact with VIRTEPEX for some time to acclimate to the system.

### Clinical Evaluation

Each participant’s strength for 4 upper extremity movements—shoulder abduction, shoulder flexion, elbow flexion, and wrist extension—was assessed by 3 different evaluators blinded to each other’s assessments. The first evaluator (a physiatrist with over 20 years of experience) conducted an in-person physical examination; the second evaluator (a medical student adequately trained in physical assessment and strength examination) conducted a synchronous standard telehealth assessment via real-time audio and video of the patient’s physical movements; and the third (another trained medical student) conducted an asynchronous VIRTEPEX assessment using the augmented reality bowling computer game alone, without corresponding audio or video of the participants’ movements. For every assessment of different joint movements, each evaluator subjectively rated patient strength on a binary scale (normal or impaired) to simplify the evaluation process and subsequent statistical analyses. In addition, the telehealth and VIRTEPEX evaluations were incorporated together into a composite evaluation that aimed to simulate a typical use-case scenario for VIRTEPEX in order to augment a typical telehealth assessment. The composite evaluation included data for wrist extension from telehealth assessments and data for shoulder abduction, shoulder flexion, and elbow flexion from VIRTEPEX assessments. This approach was formulated by the evaluator using clinical judgement to decide between data from VIRTEPEX and telehealth assessments, a process similar to using clinical judgement to extrapolate information from multiple in-person manual muscle testing data points. During evaluators’ training and interactions with VIRTEPEX, it was noted that wrist extension was identified as the most difficult upper extremity movement to capture with the Kinect camera. Therefore, the composite evaluation defaulted to telehealth assessment data for all patients’ wrist extension movements.

### Study Outcomes

Given this was a pilot study, our primary outcome was to assess for feasibility, based on participant recruitment, study completion, and safety. Recruitment rate was defined as the number of individuals who successfully enrolled and completed the study out of the total patients who were initially approached. Participants completed the study after undergoing each of the 4 upper extremity movement evaluations with each of the 3 evaluators. Safety was measured using the number of adverse events reported during VIRTEPEX evaluation.

The secondary outcome was preliminary assessment of inter-rater agreements, which were calculated for each of the 4 joint movements between (1) in-person and telehealth evaluations, (2) in-person and VIRTEPEX evaluations, (3) in-person and composite evaluations, and (4) telehealth and VIRTEPEX evaluations. Raw percent agreement between two evaluators was calculated as the number of evaluations with agreement divided by the total number of evaluations. κ statistics with 95% CI were calculated using Microsoft Excel for each joint movement to further quantify agreement between in-person and telehealth evaluations, in-person and VIRTEPEX evaluations, in-person and composite evaluations, and telehealth and VIRTEPEX evaluations. κ values were categorized on the following scale used in previous literature [28]: 0.81-1.00 (almost perfect agreement), 0.61-0.80 (excellent agreement), 0.41-0.60 (moderate agreement), 0.21-0.41 (fair agreement), 0.0-0.20 (slight agreement), and <0.00 (no agreement). Overall raw percent agreement across all joint movements was also calculated for each participant.

### Ethical Considerations

This study was approved by our institutional research ethics committee (VA Hospital Institutional Review Board, study 1643604). All participants provided written informed consent. Once evaluations were completed, data was deidentified. Participants in this project were not provided any compensation.

## Results

### User Statistics

Among 15 patients who were initially approached and agreed to participate in this study, 12 completed the evaluation, while 3 did not attend their scheduled visit. As 2 participants had bilateral upper extremity deficits and were separately evaluated for each side, there were a total of 14 completed evaluations. The study recruitment rate was 80% (N=12), with 100% of the 12 participants completing the study, and no adverse events reported.

For each evaluation method (in-person, telehealth, VIRTEPEX, and composite evaluations), 14 evaluations were conducted. This included assessments of 4 individual joint movements per evaluation, yielding 56 joint assessments per evaluation method and a total of 224 separate joint assessments across all methods. Among in-person evaluations, 38 joint assessments were rated as normal and 18 as impaired. Telehealth evaluations rated 40 joint assessments as normal and 16 as impaired. VIRTEPEX evaluations identified 37 joint assessments as normal and 19 as impaired, while composite evaluations identified 44 joint assessments as normal and 12 as impaired.

### Evaluation Outcomes

#### In-Person Versus Telehealth

Overall raw agreements for individual joint movements ranged from 71.43% to 100%, with 85.71% for shoulder abduction, 71.43% for shoulder flexion, 100% for elbow flexion, and 85.71% for wrist extension during comparison between in-person and telehealth evaluations (Table 1). κ values exhibited moderate to almost perfect agreement, ranging from κ=0.429 to κ=1.000. Substantial agreement (κ=0.720, 95% CI 0.375 to 1.000) was observed for shoulder abduction, moderate agreement (κ=0.429, 95% CI −0.025 to 0.882) for shoulder flexion, almost perfect agreement (κ=1.000, 95% CI 1.000 to 1.000) for elbow flexion, and moderate agreement (κ=0.440, 95% CI −0.155 to 1.000) for wrist extension. Based on κ values, the highest agreement was observed for elbow flexion (almost perfect with 100.00% raw agreement; κ=1.000, 95% CI 1.000 to 1.000), and the lowest for shoulder flexion (moderate agreement with 71.43% raw agreement, κ=0.429, 95% CI −0.025 to 0.882). Overall agreement between in-person and telehealth assessments was 85.71%, varying from 50% to 100% per participant.

**Table 1.** Raw percent agreements, κ statistics, and CI for in-person versus telehealth evaluations.

Variables	Joints and movements				
	Shoulder abduction	Shoulder flexion	Elbow flexion	Wrist extension	Raw agreement (%)
Research evaluation^a^					
1	A^b^	A	A	A	100
2	D^c^	A	A	A	75
3	A	A	A	A	100
4	A	A	A	A	100
5	D	D	A	A	50
6	A	D	A	D	50
7	A	A	A	A	100
8	A	D	A	A	75
9	A	A	A	A	100
10	A	A	A	D	75
11	A	A	A	A	100
12	A	A	A	A	100
13	A	A	A	A	100
14	A	D	A	A	75
Overall agreement	85.71	71.43	100	85.71	85.71
κ statistic (95% CI)	0.720 (0.375 to 1.000)	0.429 (–0.025 to 0.882)	1.000 (1.000 to 1.000)	0.440 (–0.155 to 1.000)	—^d^

a Individual participants’ responses.

b A: Agree.

c D: Disagree.

d Not applicable.

#### In-Person Versus VIRTEPEX

Overall raw agreements for individual joint movements ranged from 35.71% to 78.57%, with 57.14% for shoulder abduction, 78.57% for shoulder flexion, 78.57% for elbow flexion, and 35.71% for wrist extension during comparison between in-person evaluations and VIRTEPEX evaluations (Table 2). κ values exhibited slight to moderate agreement, ranging from κ=0.060 to κ=0.571. Fair agreement was observed (κ=0.222, 95% CI −0.073 to 0.518) for shoulder abduction, moderate agreement (κ=0.571, 95% CI 0.183 to 0.960) for shoulder flexion, fair agreement (κ=0.276, 95% CI −0.332 to 0.883) for elbow flexion, and slight agreement (κ=0.060, 95% CI −0.065 to 0.185) for wrist extension. Based on κ values, the highest agreement was observed for shoulder flexion (moderate agreement with 78.57% raw agreement; κ=0.571, 95% CI 0.183 to 0.960), and lowest for wrist extension (slight agreement with 35.71% raw agreement; κ=0.060, 95% CI −0.065 to 0.185). Overall agreement between in-person evaluations and VIRTEPEX was 62.5% , varying from 50% to 75% per participant.

**Table 2.** Raw percent agreements, κ statistics, and confidence intervals (CI) for in-person versus VIRTEPEX evaluations.

Variables	Joints and movements				
	Shoulder abduction	Shoulder flexion	Elbow flexion	Wrist extension	Raw agreement (%)
Research evaluation^a^					
1	D^b^	A^c^	A	A	75
2	D	A	A	D	50
3	A	A	A	D	75
4	A	D	A	A	75
5	D	D	A	A	50
6	D	A	D	A	50
7	A	D	A	D	50
8	D	A	A	A	75
9	A	A	D	D	50
10	A	A	A	D	75
11	A	A	A	D	75
12	A	A	A	D	75
13	A	A	D	D	50
14	D	A	A	D	50
Overall agreement (%)	57.14	78.57	78.57	35.71	62.50
κ statistic (95% CI)	0.222 (−0.073 to 0.518)	0.571 (0.183 to 0.960)	0.276 (−0.332 to 0.883)	0.060 (−0.065 to 0.185)	—^d^

a Individual participants’ responses.

b D: Disagree.

c A: Agree.

d Not applicable.

#### In-Person Versus Composite Evaluations

Overall raw agreements for individual joint movements ranged from 57.14% to 85.71%, with 57.14% for shoulder abduction, 78.57% for shoulder flexion, 78.57% for elbow flexion, and 85.71% for wrist extension during comparison between in-person and composite evaluations (Table 3). The κ values exhibited fair to moderate agreement, ranging from κ=0.222 to κ=0.571. Fair agreement (κ=0.222, 95% CI −0.073 to 0.518) was observed for shoulder abduction, moderate agreement (κ=0.571, 95% CI 0.183 to 0.960) for shoulder flexion, fair agreement (κ=0.276, 95% CI −0.332 to 0.883) for elbow flexion, and moderate agreement (κ=0.440, 95% CI −0.155 to 1.000 for wrist extension. Based on κ values, the highest agreement was observed for shoulder flexion (moderate agreement with 78.57% raw agreement; κ=0.571, 95% CI 0.183 to 0.960), and lowest for shoulder abduction (fair agreement with 57.14% raw agreement; κ=0.222, 95% CI −0.073 to 0.518). Overall agreement between in-person and composite evaluations was 75%, varying from 25% to 100% per participant.

**Table 3.** Raw percent agreements, κ statistics, and CI) for in-person versus composite evaluations.

Variables	Joints and movements				
	Shoulder abduction	Shoulder flexion	Elbow flexion	Wrist extension	Raw agreement (%)
Research evaluation^a^					
1	D^b^	A^c^	A	A	75
2	D	A	A	A	75
3	A	A	A	A	100
4	A	D	A	A	75
5	D	D	A	A	50
6	D	A	D	D	25
7	A	D	A	A	75
8	D	A	A	A	75
9	A	A	D	A	75
10	A	A	A	D	75
11	A	A	A	A	100
12	A	A	A	A	100
13	A	A	D	A	75
14	D	A	A	A	75
Overall agreement (%)	57.14	78.57	78.57	85.71	75
κ statistic (95% CI)	0.222 (−0.073 to 0.518)	0.571 (0.183 to 0.960)	0.276 (−0.332 to 0.883)	0.440 (−0.155 to 1.000)	–^d^

a Individual participants’ responses.

b D: Disagree.

c A: Agree.

d Not applicable.

#### Telehealth Versus VIRTEPEX

Overall raw agreements for individual joint movements ranged from 35.71% to 78.57%, with 71.43% for shoulder abduction, 64.29% for shoulder flexion, 78.57% for elbow flexion, and 35.71% for wrist extension during the comparison between telehealth and VIRTEPEX (Table 4). κ values exhibited poor to fair agreement, ranging from κ=−0.033 to κ=0.364. Fair agreement (κ=0.364, 95% CI −0.043 to 0.770) was observed for shoulder abduction; slight agreement (κ=0.186, 95% CI −0.342 to 0.714 for shoulder flexion, fair agreement (κ=0.276, 95% CI −0.332 to 0.883) for elbow flexion, and poor agreement (κ=−0.033, 95% CI −0.356 to 0.291) for wrist extension. Based on κ values, the highest agreement was observed for shoulder abduction (fair agreement with 71.43% raw agreement; κ=0.364, 95% CI −0.043 to 0.770), and lowest for wrist extension (poor agreement with 35.71% raw agreement; κ=−0.033, 95% CI −0.356 to 0.291). Overall agreement was 62.5%, varying from 0% to 100% per participant.

**Table 4.** Raw percent agreements, κ statistics, and CI for telehealth versus VIRTEPEX evaluations.

Variables	Joints and movements				
	Shoulder abduction	Shoulder flexion	Elbow flexion	Wrist extension	Raw agreement (%)
Research evaluation^a^					
1	D^b^	A^c^	A	A	75
2	A	A	A	D	75
3	A	A	A	D	75
4	A	D	A	A	75
5	A	A	A	A	100
6	D	D	D	D	0
7	A	D	A	D	50
8	D	D	A	A	50
9	A	A	D	D	50
10	A	A	A	A	100
11	A	A	A	D	75
12	A	A	A	D	75
13	A	A	D	D	50
14	D	D	A	D	25
Overall agreement (%)	71.43	64.29	78.57	35.71	62.5
κ statistic (95% CI)	0.364 (−0.043 to 0.770)	0.186 (−0.342 to 0.714)	0.276 (−0.332 to 0.883)	−0.033 (−0.356 to 0.291)	–^d^

a Individual participants’ responses.

b D: Disagree.

c A: Agree.

d Not applicable.

## Discussion

### Principal Results

This pilot study suggests that using VIRTEPEX to supplement telehealth strength assessment is feasible and safe. Further, VIRTEPEX demonstrates sufficiently acceptable levels of inter-rater agreement with in-person examination to warrant further evaluation for clinical use.

The inclusion of the composite assessment in this study illustrates how VIRTEPEX could be integrated into an existing clinical workflow. VIRTEPEX enables asynchronous collection of strength assessment data, which can help inform subsequent synchronous telehealth examinations. These synchronous examinations could be more efficient by focusing on movements identified as deficient via VIRTEPEX. In clinical practice, the providers could use clinical judgment to create a comprehensive assessment based on datapoints from different examination platforms. The composite evaluation in this study aims to represent this process and evaluate the utility of VIRTEPEX, and findings suggest that VIRTEPEX has the potential to augment remote strength assessments.

Among telehealth, VIRTEPEX, and composite evaluations, telehealth evaluation showed the highest overall raw agreement with in-person evaluation (85.71%). This was higher than raw percent agreements between in-person and VIRTEPEX evaluations (62.5%), in-person and composite evaluations (75%), and telehealth and VIRTEPEX evaluations (62.5%). Previous studies on inter-rater reliability for upper extremity musculoskeletal examinations have shown a wide but comparable range of agreement values. For instance, one study demonstrated raw inter-rater agreement values ranging from 66.67% to 98.9% for in-person upper extremity examinations performed by different evaluators [28], while another study comparing telemedicine-based shoulder examinations with in-person evaluations exhibited raw agreement values ranging from 46.7% to 83.7% [29].

Statistically significant agreement was observed between in-person and telehealth evaluations for shoulder abduction and elbow flexion (substantial agreement: κ=0.720, 95% CI 0.375 to 1.000 for shoulder abduction; almost perfect agreement: κ=1.000, 95% CI 1.000 to 1.000 for elbow flexion). Statistically significant, moderate agreement was observed between in-person and VIRTEPEX (κ=0.571, 95% CI 0.183 to 0.960) and in-person and composite evaluations (κ=0.571, 95% CI 0.183 to 0.960) for shoulder flexion. However, the agreement between in-person and telehealth evaluations for shoulder flexion was not statistically significant (κ=0.429, 95% CI −0.025 to 0.882). In contrast, the statistical significance of the agreements for shoulder flexion between in-person and VIRTEPEX evaluations, as well as between in-person and composite evaluations was particularly notable. These results suggest that while telehealth may have greater overall agreement with in-person evaluation and be better-suited for evaluating shoulder abduction and elbow flexion strength, VIRTEPEX and composite evaluations may be superior for assessing shoulder flexion strength in remote settings.

Interestingly, none of the remote evaluation methods demonstrated statistically significant agreement with in-person evaluations for wrist extension strength assessment. This suggests that further modifications to both existing telehealth technology and VIRTEPEX are needed to improve remote evaluation of patients with wrist weakness and/or pain. Additionally, while telehealth and VIRTEPEX evaluations showed an overall agreement of 62.5%, the lack of statistically significant κ values for the 4 joint movements suggests that the observed agreement may have been due to chance. Alternatively, it may indicate that telehealth and VIRTEPEX are better suited for assessing joint movements differently, resulting in distinct data patterns across these evaluation platforms.

### Limitations

This pilot study had several limitations. First, given that using VIRTEPEX requires reliable internet connection, this system would have limited utility for patients without internet access. Second, the sample size was small (14 evaluations) and all participants were veterans, reducing generalizability of findings to nonveteran populations. As this was a pilot study focused on feasibility, the small sample size provided less than 80% power—corresponding to approximately 22-30 evaluations—to distinguish moderate inter-rater agreement (κ=0.6) in a two-sided test [30]. A larger, fully powered study would be necessary to better quantify agreement across different evaluation methods. Additionally, VIRTEPEX is currently still under development and is not yet available for clinical practice, as further work is needed to assess and improve the system’s ease of use, cost-effectiveness, clinical outcomes, and user satisfaction. Similarly, the subjective binary strength grading used in this study was applied for simplicity and convenience during the evaluation process and statistical analyses; however, it is not applied as a standard in clinical practice.

### Future Directions

In this pilot study, VIRTEPEX was only used to remotely assess upper extremity movement strength. However, future development could enable VIRTEPEX to remotely assess strength for joint movements in other areas of the body, such as hips, knees, ankles, torso, and neck, facilitating a more comprehensive TelePA. Additional development could potentially enable VIRTEPEX to evaluate multi-joint movements, gait, and/or balance. Additionally, given that the VIRTEPEX system did not evaluate pain experienced by patients during movements, another potential feature to implement would be pain assessment.

Other potential steps could help improve appeal and adoption of VIRTEPEX. Although VIRTEPEX is currently optimized for use with Microsoft Kinect, adapting it to other devices could facilitate wider adoption. Further, streamlining the user interface and incorporating more game design elements could simplify the user experience and improve user engagement during telerehabilitation. Finally, VIRTEPEX could be expanded beyond traditional telehealth settings to other clinical applications, such as remote physiological monitoring for patients needing recurrent physical assessments.

### Conclusions

This study supports the feasibility of VIRTEPEX as a supplement to telehealth and demonstrates that VIRTEPEX can achieve moderate agreement with in-person evaluations. Notably, VIRTEPEX had greater agreement with in-person evaluations than telehealth for shoulder flexion, suggesting its potential to enhance existing telehealth technologies. Further technological developments to VIRTEPEX, combined with more adequately powered studies, can better evaluate the effectiveness and accuracy of VIRTEPEX-supplemented TelePA.

## Abbreviations

RGB-D: RGB+Depth
TelePA: tele-physical assessment
VA: Veterans Affairs
VIRTEPEX: Virtual Remote Tele-Physical Examination
